# Association Between Occupational Stress and Mental Health in Healthcare Workers During the Coronavirus Pandemic in 2019

**DOI:** 10.7759/cureus.81007

**Published:** 2025-03-22

**Authors:** Keiko Wataya, Hirokazu Tachikawa, Kiyotaka Nemoto, Shinichiro Sasahara, Yuichi Oi, Shotaro Doki, Daisuke Hori, Kokoro Hirai, Sho Takahashi, Tetsuaki Arai

**Affiliations:** 1 Department of Nursing, University of Tsukuba Hospital, Tsukuba, JPN; 2 Doctoral Program, Graduate School of Comprehensive Human Sciences, University of Tsukuba, Tsukuba, JPN; 3 Department of Disaster and Community Psychiatry, Institute of Medicine, University of Tsukuba, Tsukuba, JPN; 4 Department of Psychiatry, Institute of Medicine, University of Tsukuba, Tsukuba, JPN; 5 Occupational and Aerospace Psychiatry Group, Institute of Medicine, University of Tsukuba, Tsukuba, JPN; 6 Department of Social Mental Health, University of Tsukuba, Tsukuba, JPN; 7 Department of Psychiatry, Shibuyagawa Clinic, Tokyo, JPN; 8 Department of Medical Safety Management, University of Tsukuba Hospital, Tsukuba, JPN

**Keywords:** anxiety, covid-19, depression, occupational stress, post-traumatic stress

## Abstract

Background

During the coronavirus pandemic in 2019, deterioration of mental health was reported among healthcare workers. However, few studies have examined the relationship between healthcare workers’ mental health and occupational stress in healthcare settings during the spread of coronavirus disease 2019 (COVID-19). This study aimed to identify job stressors associated with mental health issues among healthcare workers.

Methods

The study was conducted from May 2020 to January 2021. We analyzed the impact of job stressors on mental health issues using the nine-item Patient Health Questionnaire, the seven-item Generalized Anxiety Disorder, Impact of Event Scale-Revised, and the Brief Job Stress Questionnaire in 204 healthcare workers working at a hospital treating patients with COVID-19.

Results

The results indicate that job stressors affecting depression, anxiety, and post-traumatic stress differed. High total Interpersonal Conflicts and Job Fitness stress scores were significantly associated with depressive symptoms (Interpersonal Conflicts OR:1.77; 95% CI:1.15-2.70; p = 0.001, Job Fitness OR:1.84; 95% CI:1.05-3.23; p = 0.04). High total Job Overload and Interpersonal Conflicts stress scores were significantly associated with anxiety symptoms (Job Overload OR:1.76; 95% CI:1.06-2.93; p = 0.003, Interpersonal Conflicts OR1.90; 95% CI:1.09-3.31; p = 0.002). High total Job Overload and Job Control stress scores were significantly associated with post-traumatic stress (Job Overload OR1.37; 95% CI:1.01-1.85; p = 0.04, Job Control OR1.69; 95%CI:1.17-2.43; p < 0.001).

Conclusions

These findings suggest that strategies addressing job stressors to maintain or improve mental health during an infectious disease epidemic should be tailored to individual stress responses. Additionally, the results indicate that maintaining positive interpersonal relationships in the workplace and reducing workload are particularly important for supporting mental health among healthcare workers.

## Introduction

During the coronavirus disease 2019 (COVID-19) pandemic, a deterioration of healthcare workers’ (HCWs) mental health was reported. A meta-analysis of mental health deterioration among HCWs during the COVID-19 pandemic reported a 33% prevalence of anxiety and a 28% prevalence of depression [1]. The deterioration of mental health among nurses was particularly serious. Studies have reported that being a nurse and having high anxiety are risk factors for depression [1,2]. Additionally, the risk of burnout among nurses was 4.9 times higher than among doctors [3].

Factors that have been suggested to contribute to the deterioration in HCWs’ mental health include those related to individual workplace stressors, such as a lack of infection protection supplies, insufficient human resources, workload, work environment, and a lack of professional education in infection control [4-6].

Several studies have already reported occupational stress among HCWs under pre-pandemic and pandemic conditions. Ito et al. conducted a large-scale survey of more than 9,000 HCWs in Japan before the COVID-19 pandemic and reported that the Brief Job Stress Questionnaire (BJSQ) scale scores were higher among HCWs than the national average [7]. In a previous study of physicians, job control and support from coworkers had a significant protective effect against burnout [8]. Sasaki et al. reported that the mean score of the BJSQ was higher in HCWs than in non-HCWs in a longitudinal study comparing psychological distress during the recurrence of the COVID-19 pandemic in Japan [9]. Several countries have reported a significant association between occupational stress and increased insomnia and psychological distress among healthcare workers during the COVID-19 pandemic [10,11]. However, few studies have examined the relationship between HCWs’ mental health and occupational stress in healthcare settings during the spread of COVID-19. Thus, it remains unclear which types of occupational stress affect mental health responses, such as depression, anxiety, or post-traumatic stress reactions, under pandemic conditions.

In this study, we analyzed the relationship between occupational stress and mental health among HCWs using the results of a stress check for hospital employees working in a hospital that accepted COVID-19 patients. The current study aimed to identify effective measures for reducing stress among HCWs in healthcare settings during the spread of an emerging infectious disease.

## Materials and methods

Setting and participants

We conducted an online survey of HCWs between May 6, 2020, and January 31, 2021. Nurses, physicians, and administrative staff working in an 800-bed general hospital with specific functions participated. The hospital is in the Kanto region, accepts patients with moderate to severe new coronavirus infections, and has an infectious disease ward. The survey was designed using Survey Monkey (SVMK, San Mateo, CA, USA), a web-based platform. Nurses, physicians, and administrative staff were invited to participate via a web-based bulletin board. The survey was periodically announced on the hospital's internal intranet to maximize awareness across all departments and communicated through department heads.

We estimated the required sample size using G*power 3.1.9.7 (Erdfelder et al., 1996, Heinrich-Heine University, Düsseldorf), setting parameters for multivariate logistic regression analysis. Based on a two-tailed test, a significance level of 0.05, a power of 0.95, 15 predictors, and an effect size of f2=0.15 (medium), the minimum required sample size was 199. To account for a 20% dropout rate, the target sample size was set at approximately 248 participants. The data of the respondents who agreed to participate in the survey were anonymized and analyzed.

This study included only the first response to the survey after participants began work related to COVID-19. Responses from individuals who participated multiple times for regular mental health check-ups were excluded. Furthermore, cases with more than 20% missing data in each psychological scale were examined for missing patterns and bias and excluded from the respective scale's analysis.

This study is based on mental health support initiatives conducted collaboratively by multiple departments, including clinical psychiatry, nursing, psychology, and occupational psychiatry, to protect the mental health of HCWs during the COVID-19 pandemic. Respondents who completed the stress check received feedback on their results, and those who wished were offered the opportunity to consult with mental health staff.

Measures

Sociodemographic Datasheet

The questionnaire included seven items for the sociodemographic characteristics of the medical staff: gender, age, job title, department, work status related to COVID-19, care of COVID-19-positive patients/families, and length of time since engaging in COVID-19-related work.

The 9-item Patient Health Questionnaire (PHQ-9)

The PHQ-9 is a nine-item rating scale for major depressive disorder. The Japanese version has been standardized and is used in the primary care field in Japan, with a sensitivity of 90.5% and a specificity of 76.6% for the diagnosis of major depressive disorder [12,13]. The severity of depressive symptoms in the past two weeks was reported using a four-point scale ranging from 0 to 3. The cut-off value was set at 9/10 points, suggesting major depressive disorder.

The Generalized Anxiety Disorder-7 (GAD-7)

The GAD-7 is a seven-item scale used to assess generalized anxiety disorders. Spitzer et al. developed this scale in the United States [14] and the Japanese version was validated by Muramatsu et al. [15]. The severity of anxiety symptoms in the past two weeks was reported using a 4-point scale ranging from 0 to 3. The scale measures the severity of anxiety symptoms over the past 2 weeks; a score of 0-4, 5-9,10-14, 15-19, and 20 or more indicated normal, mild, moderate, moderate to severe, and severe symptoms, respectively. The cut-off value was set at 9/10 points, suggesting a general anxiety disorder.

The Impact of Event Scale-Revised (IES-R)

The IES-R is a self-administered questionnaire developed by Weiss and Marmar [16]. The IES-R was not explicitly developed for patients with post-traumatic stress disorder. Still, it is a scale with high reliability and validity in groups exposed to traumatic stress [17]. The validity and reliability of the Japanese version of the scale were verified (Cronbach’s alpha> 0.80)[17]. In the IES-R, respondents answered 22 questions related to reexperiencing/intrusion, avoidance, and increased arousal, using a five-point scale ranging from 0 to 4. The cut-off value was set at 24/25 points, including partial post-traumatic stress disorder (PTSD). The IES-R has been used extensively with subjects who have experienced a variety of traumatic events (e.g., disasters, crimes, and bullying).

The Brief Job Stress Questionnaire (BJSQ)

The BJSQ was developed in Japan [18]. The BJSQ contains 57 items, and the Job Stressors scale includes the following nine factors: Quantitative Job Overload, Qualitative Job Overload, Physical Demand, Interpersonal Conflict, Poor Physical Environment, Job Control, Skill Discretion, Job Fitness, and Job Satisfaction. The Stress Responses scale includes Lack of Vigor, Irritability, Fatigue, Anxiety, Depressed Mood, and Somatic Symptoms. The Social Supports scale includes Supervisor Support, Coworker Support, Family Support, and Life-Job Satisfaction. The questionnaire was based on the National Institute for Occupational Safety and Health (NIOSH) occupational stress model, which has been reported to have acceptable reliability in previous studies (Cronbach’s alpha>0.70) [19]. The BJSQ has a limitation in that its concurrent validity has not been thoroughly examined against globally recognized occupational stress scales with established reliability and validity [19]. However, given its widespread use in previous studies and across various occupations and workplaces in Japan, we considered it valuable for comparative analysis in this study. Responses were derived from self-administered questionnaires using a 4-point Likert scale (1. very much agree - 4. completely disagree). The determination of the highly stressed status was conducted following the implementation manual prepared by the Japanese Ministry of Health, Labour, and Welfare [20]. A person was judged to be highly stressed if the total score of mental and physical stress responses was 77 points or more if the total score of job stressors and support from surroundings was 76 points or more, and if the total score of stress responses was 63 points or more. In simplified scoring, stressors are classified into five factors and two stress reactions. Job stressors include Job Overload, Job Control, Interpersonal Relationships, and Job Fitness. Stress responses include a Lack of Vigor, Irritability, Fatigue, Anxiety, Depressed Mood, and Somatic Symptoms. Social supports include Supervisor Support, Coworker Support, and Family Support. Details of job stressors are as follows: Job Overload consists of Quantitative Job Overload (e.g., I have an extremely large amount of work to do), Qualitative Job Overload (e.g., I have to pay very careful attention), and Physical Demand (e.g., My job requires a lot of physical work). Other job stressors are Job Control (e.g., I can reflect on my opinions on workplace policy), Interpersonal Relationships (e.g., the atmosphere in the workplace is friendly), and Job Fitness (e.g., this job suits me well).

The non-open-access Japanese version of the PHQ-9 and the Japanese version of the GAD-7 were used with permission from the developers.

Statistical Analysis

We first compared the baseline characteristics of those who did and did not have depression, anxiety, post-traumatic stress symptoms, and high job stress using a chi-squared test for categorical variables and the Mann-Whitney U test for continuous variables. Multivariate logistic regression analysis was used to identify possible occupational stressors related to depression, anxiety, and post-traumatic stress symptoms. Continuous predictor variables included Age, Job Overload, Job Control, Interpersonal Conflict, Job Fitness, and Social Support. Categorical variables included gender, occupation type (doctors/nurses), and department (infectious disease ward/others). Associations between risk factors and outcomes are presented as odds ratios (OR) and 95% confidence intervals (CI). For the stress scale scores on the BJSQ, the Stress Response was excluded to account for multicollinearity in the regression analysis. All analyses were performed using SPSS statistical software version 24.0 (IBM Corp., Armonk, NY, US), with a two-tailed significance test set at p .05.

## Results

Characteristics of participants

Demographic characteristics are shown in Table 1. Of the 2,490 workers invited to the survey, 204 (8.1%) participated. Regarding profession, 146 (71.6%) participants were nurses, and 58 (28.4%) were doctors, medical staff, and administrative staff. Regarding gender, 48 (23.5%) participants were male. Regarding age, 112 (54.9%) participants were under 40 years. One hundred five (105) participants (51.1%) had been involved in COVID-19-related duties for more than 4 weeks. A total of 132 respondents (68.0%) belonged to departments that accepted COVID-19-positive patients.

**Table 1:** Demographic characteristics of the participants ^a ^Pharmacologist, technician, social worker, psychologist, therapist, nutritionist ^b^ Infectious disease units, intensive care units, and emergency rooms are directly involved in the diagnosis, treatment, and care of patients with or suspected of having COVID-19 ^c^ General inpatient ward, rehabilitation department, nutrition department, administrative department

Variables	Count (%)
Workers	2490
Participants, n	204 (8.1)
Gender, n (%)	
Males	48 (23.5)
Females	156 (76.5)
Age group, n (%)	
18-39 years	112 (54.9)
Role, n (%)	
Doctors	26 (12.7)
Nurses	146 (71.6)
Other medical staff ^a^	17 (8.3)
Administrative staff	15 (7.4)
Medical setting, n (%)	
Frontline department ^b^	132 (68.0)
Other practice locations ^c^	62 (32.0)
No responses	10
Period of work (related to COVID-19), n (%)	
More than 4 weeks	105 (51.5)
Not applicable	11 (5.4)

Status of depression, anxiety, stress disorder, and job stress

Analysis of each psychological test score and attribute is shown in Table 2. Among the participants, 21.1% were above the cut-off score on the PHQ-9, indicating possible depression, and 12.3% were above the cut-off score on the GAD-7, suggesting possible anxiety. Additionally, 24.5% scored above the IES-R cut-off for PTSD. The proportion of individuals aged 18-39 years, those who were nurses, and those who had worked for less than 4 weeks was significantly higher in the depression group than in the healthy group (χ2 (1) = 3.95, 6.50, 6.76; p= 0.047, 0.011, 0.034 for each). The proportion of participants who worked for less than 4 weeks was significantly higher in the anxiety group (5 (2.9%), χ2 (1) = 12.72, p = 0.002). Additionally, the proportion of participants aged 18-39 years was significantly higher in the PTSD group (χ2 (1) = 9.58, p = 0.002).

**Table 2:** Mental health measurements in the total cohort and subgroups (n=204) *P< .05 **P < .01 ^a^ Doctors, pharmacologists, technicians, social workers, psychologists, therapists, nutritionists ^b^ Infectious disease units, intensive care units, and emergency rooms are directly involved in the diagnosis, treatment, and care of patients with or suspected of having COVID-19 Abbreviations: PTSD, post-traumatic stress disorder; PHQ-9, 9-item Patient Health Questionnaire; GAD-7, 7-item Generalized Anxiety Disorder Scale; IESR, Impact of Event Scale-Revised; N/A, not applicable

Variables	PHQ-9<10	PHQ-9≥10	χ2	p-value	GAD-7<10	GAD-7≥10	χ2	p-value	IES-R < 24	IES-R≥24	χ2	p-value
Overall	161 (78.9)	43 (21.1)	N/A	N/A	179 (87.7)	25 (12.3)	N/A	N/A	154 (75.5)	50 (24.5)	N/A	N/A
Gender												
Males	43 (21.1)	5 (2.5)	4.29	0.04*	43 (21.1)	5 (2.5)	0.20	0.66	40 (19.6)	8 (3.9)	2.09	0.15
Females	118 (57.8)	38 (18.6)			136 (66.7)	20 (9.8)			114 (55.9)	42 (20.6)		
Role												
Nurses	110 (53.9)	36 (17.6)	3.95	0.05*	125 (61.3)	21 (10.3)	2.16	0.14	107 (52.5)	39 (19.1)	1.35	0.25
Others ª	51 (25.0)	7 (3.4)			54 (26.5)	4 (2.0)			47 (23.0)	11 (5.4)		
Age group												
18-39 years	81 (39.7)	31 (15.2)	6.5	0.01*	92 (45.1)	20 (9.8)	2.16	0.14	75 (36.8)	37 (18.1)	9.58	0.00**
40 years and older	80 (39.2)	12 (5.9)			87 (42.6)	5 (2.5)			79 (38.7)	13 (6.4)		
Medical setting												
Frontline department ^b^	104 (53.6)	28 (14.4)	0.00	0.97	115 (59.3)	17 (8.8)	0.97	0.32	102 (52.6)	30 (15.5)	0.05	0.82
Others	49 (25.3)	13 (6.7)			57 (29.4)	5 (2.6)			47 (24.2)	15 (7.7)		
Period of work												
Within 4 weeks	62 (30.4)	26 (12.7)	6.76	0.03*	69 (33.8)	19 (9.3)	12.72	0.00*	62 (30.4)	26 (12.7)	3.74	0.16
More than 4 weeks	90 (44.1)	15 (7.4)			100 (49.0)	5 (2.5)			85 (41.7)	20 (9.8)		

A comparison of the scores for each job stressor among depression, anxiety, and PTSD is shown in Table 3. The values were significantly higher in the depression group than in the healthy group. Apart from social support, the scores were significantly higher in the anxiety and post-traumatic stress groups than in the healthy group.

**Table 3:** Comparison of job stressors and mental health measurements at the cutoff point of the scale *P< .05; **P < .01 ^a^ This score includes Quantitative Job Overload, Qualitative Job Overload, and Physical Demand. ^b ^This score includes Supervisor Support and Coworker Support. Abbreviations: PTSD, post-traumatic stress disorder; PHQ-9, 9-item Patient Health Questionnaire; GAD-7, 7-item Generalized Anxiety Disorder Scale; IESR, Impact of Event Scale-Revised; IQR, interquartile range

Variables	PHQ-9<10	PHQ-9≥ 10	p-value	GAD-7<10	GAD-7≥10	p-value	IES-R<24	IES-R≥24	p-value
Job Overload ^a^, median (IQR)	6 (5-7)	6 (6-7)	0.01*	6 (5-7)	7 (6-7)	0.00**	6 (5-7)	6 (6-7)	0.00**
Job Control, median (IQR)	1 (0-2)	2 (1-3)	0.01*	1 (0-2)	2 (1-3)	0.04*	1 (0-2)	2 (1-3)	0.00**
Interpersonal Conflict, median (IQR)	0 (0-1)	1 (0-2)	0.00**	0 (0-1)	1 (0.5-2)	0.00**	0 (0-1)	1 (0-2)	0.00**
Job Fitness, median (IQR)	0 (0-1)	1 (0-1)	0.00**	0 (0-1)	1 (0-1-5)	0.01*	0 (0-1)	1 (0-1)	0.00**
Social Supports ^b^, median (IQR)	3 (1-5)	4 (3-6)	0.02*	3 (1-5)	3 (1.5-5)	0.65	3 (1-5)	3 (1-5)	0.41

Risk factors for depression, anxiety, and post-traumatic stress

The potential risk factors for depression, anxiety, and post-traumatic stress were examined using multivariate logistic regression analysis, as shown in Table 4. Multicollinearity was assessed by confirming that all explanatory variables' variance inflation factor values were below 5.

**Table 4:** Risk factors for depression (total PHQ-9 score ≥10), anxiety (total GAD-7 score ≥10), and post-traumatic stress (total IES-R score ≥24) by multivariable logistic regression analysis *P< .05 **P < .01 ^a^ Doctors, pharmacologists, technicians, social workers, psychologists, therapists, nutritionists, administrative staff ^b^ General inpatient ward, rehabilitation department, nutrition department, administrative department ^c^ Infectious disease units, intensive care units, and emergency rooms are directly involved in the diagnosis, treatment, and care of patients with or suspected of having COVID-19 ^d^ This score includes Quantitative Job Overload, Qualitative Job Overload, and Physical Demand. ^e^ This score includes Supervisor Support and Coworker Support. Abbreviations: PTSD, post-traumatic stress disorder; PHQ-9, 9-item Patient Health Questionnaire; GAD-7, 7-item Generalized Anxiety Disorder Scale; IESR, Impact of Event Scale-Revised; OR, odds ratio; CI, confidence interval; VIF, variance inflation factor; ref., reference

Variables	Depression		Anxiety		PTSD		-
-	OR (95%CI)	P-value	OR (95%CI)	P-value	OR (95%CI)	P-value	VIF
Gender (ref. Males)							
Females	2.62 (0.76-9.01)	0.13	0.90 (0.23-3.52)	0.87	1.57 (0.54-4.60)	0.41	1.13
Age group (ref. 40 years and older)							
18-39 years	2.17 (0.95-4.96)	0.07	3.84 (1.14-12.91)	0.03*	3.01 (1.32-6.86)	0.01*	1.10
Occupation (ref. Others ^a^)							
Nurses	1.53 (0.48-4.92)	0.47	1.61 (0.292-8.85)	0.59	0.64 (0.22-1.85)	0.41	1.42
Department (ref. Others ^b^)							
Frontline department ^c^	0.88 (0.36-2.14)	0.77	1.73 (0.52-5.74)	0.37	0.71 (0.30-1.69)	0.44	1.09
Job stressors							
Job Overload score^d^	1.30 (0.96-1.77)	0.09	1.76 (1.06-2.93)	0.03*	1.37 (1.01-1.85)	0.04*	1.21
Job Control score	0.95 (0.65-1.38)	0.77	1.02 (0.63-1.64)	0.94	1.69 (1.17-2.43)	0.00**	1.30
Interpersonal Conflict score	1.77 (1.15-2.70)	0.01*	1.90 (1.09-3.31)	0.02*	1.44 (0.95-2.16)	0.09	1.08
Job Fitness score	1.84 (1.05-3.23)	0.04*	1.70 (0.83-3.50)	0.15	1.46 (0.84-2.51)	0.18	1.22
Social Support score ^e^	1.19 (0.98-1.43)	0.07	1.02 (0.81-1.29)	0.88	0.98 (0.82-1.18)	0.85	1.07

High total Interpersonal Conflicts and Job Fitness stress scores were significantly associated with depressive symptoms (Interpersonal Conflicts OR: 1.77; 95% CI: 1.15-2.70; p = 0.001, Job Fitness OR: 1.84; 95% CI: 1.05-3.23; p = 0.04). High total Job Overload and Interpersonal Conflicts stress scores were significantly associated with anxiety symptoms (Job Overload OR:1.76; 95% CI:1.06-2.93; p = 0.003, Interpersonal Conflicts OR1.90; 95% CI:1.09-3.31; p = 0.002). High total Job Overload and Job Control stress scores were significantly associated with post-traumatic stress (Job Overload OR 1.37; 95% CI:1.01-1.85; p = 0.04, Job Control OR 1.69; 95% CI:1.17-2.43; p < 0.001). Moreover, participants who were 18-39 years old had significantly more symptoms of anxiety and post-traumatic stress than those who were 40 years or older (OR:3.84; 95% CI:1.14-12.91; p =0.03 and OR:3.01; 95% CI:1.32-6.86; p = 0.01).

## Discussion

This study aimed to explore the relationship between occupational stressors and stress responses among HCWs during the early stages of an emerging infectious disease outbreak. The interpretation of each result is discussed below.

Characteristics of stress responses

In our study, the proportion of participants with depressive symptoms was greater than that of participants with anxiety symptoms, which is consistent with previous studies [3,21,22]. A strong tendency for post-traumatic stress in the initial stages of the COVID-19 epidemic has been reported worldwide due to the fear of an unknown virus and lack of protective equipment [21]. This trend was also observed in the current survey, which was conducted during the same period. A meta-analysis of previous studies reported a pooled prevalence of depression of 21.7% (95% CI, 18.3-25.2%), anxiety of 22.1% (95% CI, 18.2-26.3%), and PTSD of 21.5% (95% CI, 10.5-34.9%) among HCWs during the COVID-19 pandemic [22]. These differences in prevalence have been attributed to bias in sampling methods, regional differences, and measurement tools. Nevertheless, it has been suggested that HCWs are at risk of developing common mental disorders during pandemics [22]. Furthermore, the trends of general deterioration and regional differences in stress are similar to those observed during natural disasters [23]. During the survey period, a state of emergency was declared, medical institutions that accepted positive patients were required to implement infection prevention measures, and a new infectious disease ward was established. Therefore, HCWs had to change their duties and adapt to new work environments and relationships. Subsequently, stress reactions tended to be higher among younger age groups in this study. In a previous study, Jose et al. reported that young people who reported higher levels of social connectedness at one point in time subsequently reported better well-being [24]. However, to prevent infection during the COVID-19 pandemic, it was necessary to reduce social contact. This social environment may have had a significant impact on the mental health of younger HCWs.

In the current survey, the occupation with the highest number of respondents was nurses. This study did not find an association between nurses and anxiety or stress symptoms, but nurses may be more associated with depressive symptoms than other professionals (p=.047). In previous studies, the worsening of depressive symptoms was reported in nurses during the COVID-19 outbreak [3,25]. During the COVID-19 pandemic, several qualitative studies have found that a sense of uncertainty lingered among nurses and that unfamiliar tasks and environments generated physical and psychological difficulties for them [25]. It has been suggested that the stress of facing multiple complex tasks may be the cause of nurses' depressive symptoms.

Characteristics of occupational stressors that affected the stress response

In the regression analysis, various job stressors were found to influence depression, anxiety, and post-traumatic stress reactions among HCWs. First, Interpersonal Conflicts and Job Fitness had a significant influence on the depression group. Worsening depressive symptoms among HCWs during the COVID-19 pandemic have been reported [2,6]. Rapid changes in workplace and external conditions due to the pandemic response were suggested to have increased stress related to interpersonal conflicts and job suitability, contributing to the exacerbation of depressive symptoms. These findings suggest that addressing depressive symptoms during emerging infectious disease outbreaks requires interventions to manage changes in interpersonal relationships resulting from modifications in the work environment and job locations. Previous studies on nurses have reported that mutually supportive relationships with colleagues provide encouragement and reassurance [26]. Therefore, stress reduction measures, such as Job Aptitude and Interpersonal Conflicts, should be considered during outbreaks of infectious diseases that result in departmental transfers or changes in job content. Specifically, it is crucial to clarify consultation roles within a department.

Second, Age, Job Overload, and Interpersonal Conflict significantly influenced the anxiety group. A previous study reported the physical burden of wearing protective gear [27] and higher rates of insomnia among HCWs during the COVID-19 pandemic than among the general population [28]. It is important to reduce Job Overload among individuals by considering the nature of the work environment, such as night shift duties, and ensuring the availability of rest periods to reduce anxiety. Previous research has reported that individuals in their 40s experience greater anxiety and fatigue than those in their 20s [29]. This finding differed from our results, in which younger generations were more affected by anxiety. This discrepancy may reflect the specific timing and setting of our study, where healthcare workers in their 20s and 30s experienced frequent ward transfers and job changes. Given that anxiety levels may vary across age groups depending on when a study is conducted, it is essential to consider the characteristics and phases of an infectious disease outbreak, as well as the duration of countermeasures, to better identify high-risk populations. Additionally, Interpersonal Conflict was a common influencing factor for depressive symptoms, suggesting that relationship-related stress contributed to various forms of psychological distress. Previous studies have emphasized that chatting with family and colleagues and receiving positive attitudes from coworkers are important ways to cope with stress during the COVID-19 pandemic [30]. These findings made interventions to facilitate smoother interpersonal relationships vital under pandemic conditions. Therefore, peer support among staff in the same situation and sharing notes within the department may also be an effective way to reduce stress and anxiety.

Finally, Age, Job Overload, and Job Control significantly influenced the post-traumatic stress group. A study conducted during the Severe Acute Respiratory Syndrome (SARS) outbreak reported that increased workload and conflicts among colleagues were strongly associated with traumatic stress responses [31]. Similarly, our findings indicated that job overload influenced traumatic stress responses. Additionally, this study found that job control also had an impact on traumatic stress responses. Previous research has reported that frontline nurses experienced psychological distress while coping with work demands and social relationships during the global spread of COVID-19 [26]. This suggests that changes in lifestyle and job responsibilities may have led to a loss of a sense of control. Greater attention should be given to the stress experienced by healthcare providers due to job changes, and managerial considerations that incorporate individual opinions and preferences in work arrangements may help prevent traumatic stress responses.

These results suggest that different approaches can maintain and improve mental health depending on an individual’s stress response. However, multiple occupational stressors have aggravated the mental health of HCWs. Therefore, it is again suggested that maintaining mental health during the early stages of the pandemic requires a combination of different types of support that have been recommended.

Limitations

This survey was conducted at a single facility during the early stages of the COVID-19 pandemic. It was a voluntary online survey, which may have contributed to a sample size smaller than the target. As a result, there were limitations in describing the mental health of all employees overall in a medical setting during the COVID-19 pandemic. Additionally, since the survey was part of a mental health checkup for the staff, items related to private information were kept minimal to avoid the risk of identifying individuals. Therefore, psychosocial questions, such as those regarding participants' history of mental illness, were not included. Due to this limitation, a ceiling effect may have occurred, leading to an overestimation of mental health deterioration. Furthermore, the severity of the patient's illnesses and the duration of the HCWs’ involvement were not clearly defined. The study also used convenience sampling, which may have led to selection bias, particularly as staff from departments most affected by the COVID-19 pandemic might have been overrepresented. While there are several limitations, this study is one of the few reports focusing specifically on the relationship between occupational stressors and stress responses arising from the COVID-19 pandemic. It will provide insights into healthcare worker health management during the emergence of new infectious diseases and disasters. In the future, we need to conduct a more detailed survey on the mental health of HCWs by devising a research design and increasing the number of participants. A more detailed examination of the factors that contribute to mental health decline is required.

## Conclusions

We conducted a stress survey of employees at a hospital that treated COVID-19 patients. The results revealed that the prevalence of depression, anxiety, and post-traumatic stress was similar to that in previous studies in Japan. The occupational stressors that affected depression, anxiety, and post-traumatic stress were different. Thus, the current results suggest that approaches to occupational stress, which are associated with stress reactions, may be useful for addressing depression, anxiety, and post-traumatic stress. In addition, younger participants were more affected by anxiety and post-traumatic stress than depression. Therefore, psychosocial support for Interpersonal Conflict and Job Control, which are occupational stressors that influence anxiety and post-traumatic stress, is suggested to be important for supporting young people. To maintain the mental health of healthcare workers during the spread of the COVID-19 pandemic, hospital organizations needed to consider the work environment. Additionally, it is important to provide psychological and social support (e.g., peer support groups and daily debriefings) in similar disaster situations.
